# Prevention of gestational diabetes in pregnant women with obesity: protocol for a pilot randomised controlled trial

**DOI:** 10.1186/s40814-022-01021-3

**Published:** 2022-03-25

**Authors:** Ola F. Quotah, Glen Nishku, Jessamine Hunt, Paul T. Seed, Carolyn Gill, Anna Brockbank, Omoyele Fafowora, Ilektra Vasiloudi, Opeoluwa Olusoga, Ellie Cheek, Jannelle Phillips, Katarzyna G. Nowak, Lucilla Poston, Sara L. White, Angela C. Flynn

**Affiliations:** 1 Department of Women and Children’s Health, School of Life Course Sciences, King’s College London, 10th Floor North Wing, St Thomas’ Hospital, Westminster Bridge Road, London, SE1 7EH UK; 2 Department of Clinical Nutrition, Faculty of Applied Medical Science, King Abdulaziz University, Jeddah, Kingdom of Saudi Arabia; 3 Department of Nutritional Sciences, School of Life Course Sciences, King’s College London, Franklin-Wilkins Building, 150 Stamford Street, London, SE1 9NH UK

**Keywords:** Gestational diabetes, Maternal obesity, Lifestyle intervention, Metformin

## Abstract

**Background:**

Obesity in pregnancy increases the risk of gestational diabetes mellitus (GDM) and associated adverse outcomes. Despite metabolic differences, all pregnant women with obesity are considered to have the same risk of developing GDM. Improved risk stratification is required to enable targeted intervention in women with obesity who would benefit the most. The aim of this study is to identify pregnant women with obesity at higher risk of developing GDM and, in a pilot randomised controlled trial (RCT), test feasibility and assess the efficacy of a lifestyle intervention and/or metformin to improve glycaemic control.

**Methods:**

Women aged 18 years or older with a singleton pregnancy and body mass index (BMI) ≥ 30kg/m^2^ will be recruited from one maternity unit in London, UK. The risk of GDM will be assessed using a multivariable GDM prediction model combining maternal age, mid-arm circumference, systolic blood pressure, glucose, triglycerides and HbA1c. Women identified at a higher risk of developing GDM will be randomly allocated to one of two intervention groups (lifestyle advice with or without metformin) or standard antenatal care. The primary feasibility outcomes are study recruitment, retention rate and intervention adherence and to collect information needed for the sample size calculation for the definitive trial. A process evaluation will assess the acceptability of study processes and procedures to women. Secondary patient-centred outcomes include a reduction in mean glucose/24h of 0.5mmol/l as assessed by continuous glucose monitoring and changes in a targeted maternal metabolome, dietary intake and physical activity. A sample of 60 high-risk women is required.

**Discussion:**

Early risk stratification of GDM in pregnant women with obesity and targeted intervention using lifestyle advice with or without metformin could improve glucose tolerance compared to standard antenatal care. The results from this feasibility study will inform a larger adequately powered RCT should the intervention show trends for potential effectiveness.

**Trial registration:**

This study has been approved by the NHS Research Ethics Committee (UK IRAS integrated research application system; reference 18/LO/1500). EudraCT number 2018-000003-16.

## Introduction

Global estimates suggest that over 21% of women will be obese by 2025 [1]. In the UK, over 20% of women present with obesity at their first antenatal visit [2]. Obesity adversely affects reproductive health [3]. Pregnant women with obesity have a higher risk of developing most pregnancy complications, including gestational diabetes mellitus (GDM) [4, 5] and women with GDM are more likely to develop hypertension and pre-eclampsia during pregnancy [6, 7] as well as cardiovascular disease and type 2 diabetes later in life [8–10]. Infants born to mothers with GDM are more likely to be born large-for-gestational age (LGA) and macrosomic, increasing the risk of complications at delivery, including shoulder dystocia [7, 11]. Furthermore, offspring of GDM mothers have a higher risk of being overweight or obese [12], developing diabetes [13, 14] or metabolic disease [15, 16] later in life.

UK guidelines recommend that all pregnant women with obesity undergo an oral glucose tolerance test (OGTT) at 24–28 weeks’ gestation to detect GDM and introduce treatment [17]. Evidence suggests, however, that excessive fetal growth precedes the time of diagnosis in obese women [18]. Furthermore, marked abnormalities in the metabolic profiles of obese women who develop GDM may occur at least 10 weeks before conventional diagnosis [19, 20].

Several randomised controlled trials (RCTs) have attempted to prevent adverse outcomes in obese women; diet and physical activity [21] or pharmacological interventions [22–24] have overall not been effective. This has shifted the focus to targeted intervention for those individuals identified at greatest risk and increasing evidence suggests that this approach might be beneficial. The RADIEL RCT of a lifestyle intervention in 293 Finnish pregnant women, of whom 30% had previous GDM, demonstrated a reduction in GDM from 21.6 to 13.9% [25]. This observation was further supported by a UK multicentre randomised trial (ESTEEM) of a Mediterranean-style diet in women with risk factors including obesity, chronic hypertension or hypertriglyceridaemia, in which a reduction in GDM of 35% in the intervention group was achieved [26].

Although several studies describe tools developed to predict GDM in weight heterogenous women [27–33], specific approaches for pregnant women with obesity are rare. At present, all pregnant women with a body mass index (BMI) ≥ 30 kg/m^2^ are considered at high risk of developing GDM, although the majority do not develop the condition [34]. Our prediction tool for GDM was constructed using a range of biochemical and clinical factors in early pregnancy obtained in a cohort of 1303 obese pregnant women who took part in the UK Pregnancies Better Eating and Activity Trial (UPBEAT) [34]. The tool was developed using the clinical variables maternal age, blood pressure and mid-arm circumference and the biomarkers HbA1c, glucose and triglycerides. At a threshold of ≥ 35% risk, the tool identifies in early pregnancy obese pregnant women with a higher risk of developing GDM (1 in 2 chance) when diagnosed using The International Association of Diabetes and Pregnancy Study Groups (IADPSG) criteria and thereby those who might benefit most from an intervention [35].

In this, the next step, a pilot study will be undertaken in which pregnant women with obesity and at higher risk of developing GDM as identified using the prediction tool will be randomised to one of two interventions designed to improve glucose tolerance or to standard antenatal care.

### Study objectives

The objectives of the UK Pregnancies Better Eating and Activity Trial-Taking It Forward (UPBEAT-TIF) study are:

1. *Primary feasibility outcomes*: To evaluate the feasibility (recruitment, retention, intervention adherence, determination of sample size) and acceptability of the UPBEAT-TIF intervention in women who are identified as having a higher risk of developing GDM
2. *Secondary patient-centred objectives*

- To assess in pregnant women with obesity, identified as high risk for GDM, the efficacy of (a) lifestyle advice (diet and physical activity) and (b) metformin treatment plus lifestyle advice, to improve maternal glycaemic control, when compared with (c) standard care
- To determine the impact of each intervention on a targeted maternal metabolome
- To examine the effect of each intervention on dietary intake and physical activity

## Methods and analysis

This protocol paper is written in accordance with the Standard Protocol Items: Recommendations for Interventional Trials (SPIRIT) checklist [36].

**Fig. 1:**
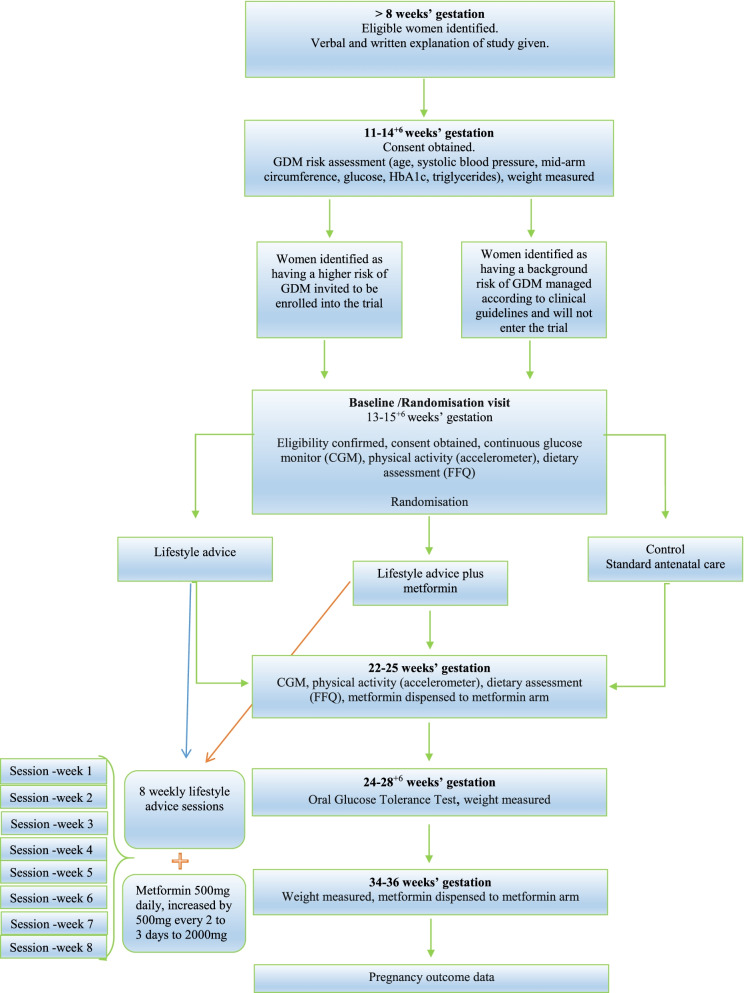
CONSORT study flow diagram for the UPBEAT-TIF study

This study will be a single-centre, open-label, randomised controlled trial. NHS Research Ethics Committee approval has been obtained (UK IRAS integrated research application system; reference 18/LO/1500). Women will be recruited in one centre, from the antenatal clinics of Guy’s and St Thomas’ NHS Foundation Trust, London, UK (Fig. 1). Pregnant women with obesity will be invited to be screened for risk of developing GDM at their routine nuchal ultrasound scan appointment at St Thomas’ Hospital. Women will be eligible to take part in the study if they meet the following criteria:

- Over 18 years
- BMI ≥ 30kg/m^2^
- Higher risk of GDM as identified by risk assessment at screening
- Singleton pregnancy
- Gestation 11–14^+6^ weeks’ at the time of screening
- Willing and able to give written informed consent

Women will be excluded if they meet the following criteria:

- Women identified as being at background risk of GDM at screening
- Taking metformin or any medications that affect insulin sensitivity
- HbA1c ≥6.5% at the time of screening
- Pre-existing medical conditions including diabetes, thyroid disease, coeliac disease, hypertension, sickle cell, systemic lupus erythematosus, antiphospholipid syndrome, thalassaemia and current psychosis
- Past bariatric surgery
- Multiple pregnancy
- Insufficient understanding of the trial
- Contraindications to metformin
- Participation in another investigational medicinal product trial at the time of screening

### Study entry

#### 11–14^+6^ weeks’ gestation appointment: screening

Eligible women will be contacted by a clinical research practitioner/assistant and offered information about the study before their routine nuchal ultrasound scan appointment in the antenatal clinic of St Thomas’ Hospital. Eligible women will also be approached in the antenatal clinic, given information about the study and offered the prediction test to identify the risk of later GDM development. Following informed written consent, weight will be measured and a blood sample taken at the same time as routine blood sampling for measurement of HbA1c, glucose and triglycerides. Clinical risk factors will also be recorded (age, blood pressure, mid-arm circumference) for risk assessment. Risk will be assessed by the algorithm previously described [35].

All women screened will be contacted by telephone. Women identified as being at background risk of developing GDM will be managed according to clinical guidelines and will not enter the trial. Women identified as being at a higher risk will be invited to be randomised into an antenatal programme of lifestyle advice, lifestyle advice plus metformin treatment or standard antenatal care.

#### 13–15^+6^ weeks’ gestation appointment: baseline and randomisation

At the first appointment, informed consent will be obtained by the chief investigator from women to be randomised into the trial. All participants will be fitted with a glucose sensor (Dexcom G6) to be worn for 7–10 days. The sensors are small, easy to wear, waterproof devices which require no finger prick calibration. The sensors will be applied to the back of the upper arm or abdomen of the participant and activated in blinded mode. To assess dietary intake, a semi-quantitative food frequency questionnaire (FFQ) will be administered to evaluate dietary glycaemic load (GL), glycaemic index (GI), saturated fat and other dietary variables [34]. Physical activity will be assessed by Axivity accelerometer AX3 (https://axivity.com/product/ax3).

Following the baseline assessment, randomisation will be performed electronically by the research team via a secure online-based data management platform (MedSciNet™) in order to conceal the sequence until interventions are assigned. Women will be randomised (1:1:1) into three groups:

a. Lifestyle advice

b. Lifestyle advice plus metformin

c. Standard antenatal care

The randomisation schedule will be minimised according to ethnicity (Black, White, Asian, Other), BMI (30−34.9kg/m^2^, 35−39.9kg/m^2^, ≥40kg/m^2^), parity (nulliparous/multiparous) and age. Participants will be allocated sequential study numbers regardless of allocation to the intervention or standard care group. Due to the nature of the study design, it will not be possible to blind participants or research staff to the randomisation arm.

#### Intervention

##### Lifestyle advice

Participants allocated to the lifestyle advice or lifestyle advice plus metformin arms will receive the intervention. The dietary intervention, delivered by a nutritionist, focuses on reducing GL and saturated fat intake using a regime previously shown to be effective in changing diet in ‘all risk’ obese pregnant women [34]. The dietary component of the intervention will aim to promote a healthier pattern of eating without restricting energy intake. The dietary advice will be tailored according to the woman’s habitual diet and cultural preference.

To decrease GL, participants will be encouraged to exchange starchy foods with a medium to high GI for those with a lower GI and reduce the consumption of sugar-sweetened beverages including fruit juice. Specific dietary goals will be provided for each participant that focus on differentiating between carbohydrate-rich foods. This advice will include replacing high GI breads with lower GI breads such as granary/wholegrain/multigrain bread, sourdough and rye; replacing high GI breakfast cereals with lower GI varieties such as porridge, oat-based cereals and no-added-sugar muesli; and encouraging consumption of lower GI grains like basmati rice, pearl barley, cracked wheat (bulgur), quinoa and pasta. To reduce saturated fat intake, participants will be encouraged to swap oils and spreads high in saturated fats (butter, lard, ghee) for those high in mono- and polyunsaturated fats (olive, sunflower); swap meat high in saturated fat (fatty meats, processed meat and meat products) for lean red meat, chicken and fish; and swap higher fat dairy foods (milk, cheese, yogurt) for lower fat varieties.

The physical activity component of the intervention will focus on a safe physical activity regime using the UPBEAT strategy which was shown to be successful in improving physical activity in ‘all risk’ pregnant women with obesity [34]. The advice aims to increase incremental walking and daily step count at a moderate intensity, being more active in daily life and reducing sedentary behaviour (sitting and screen time).

##### Metformin

In participants randomised to the lifestyle intervention plus metformin arm, metformin treatment will be initiated (500mg daily with food) and increased by 500mg every 2 to 3 days to achieve 2000mg within 2 weeks, taken as divided doses. Should side-effects occur, participants will be advised to reduce the current dose to that of the previous week and wait for 1 week before increasing the dose again. Treatment will be initiated after randomisation and continued until the delivery of the baby. Metformin will be dispensed at study visits and adherence assessed by tablet count.

Participants randomised to the lifestyle intervention arms will receive weekly sessions for 8 consecutive weeks. The sessions will be covered by video, email or telephone. Following a general review of the lifestyle or lifestyle plus metformin intervention, each session will be designed to deliver a different element of the intervention. The development of the UPBEAT lifestyle intervention was informed by psychological models of health behaviour, including control and social cognitive theory [34]. Participants will be encouraged to set achievable goals using the SMART (Specific, Measurable, Achievable, Relevant, Time Specific) goal approach, which will be reviewed on a weekly basis. In addition, participants will be asked to self-monitor their progress using a logbook. The participants will also receive a handbook with guidance on making the changes, along with recipe ideas and general information on eating while pregnant.

##### Standard care

Women randomised to the standard care group will attend all study visits except the 8 weekly intervention sessions and receive routine antenatal care according to local health care provision.

#### 22–25 weeks’ gestation appointment

All participants will be fitted with a continuous glucose monitoring (CGM) sensor for 7–10 days, again in blinded mode. Dietary intake and physical activity will be assessed using the FFQ and accelerometer. In the ‘lifestyle plus metformin’ arm, metformin will be dispensed, information on any side effects or adverse events will be collected and adherence will be assessed by tablet count.

#### 24–28 weeks’ gestation appointment

All participants will be asked to attend at 24–28 weeks’ gestation for an oral glucose tolerance test (fasting for a minimum of 10 h, 75 g glucose load). Routine clinical blood samples will be taken fasted and 2 h after the glucose load in accordance with The National Institute of Clinical Excellence (NICE) guidelines for the diagnosis of GDM [17]. Research blood samples will be obtained in the fasted state, 1 h and 2 h following the glucose load. For the purposes of the research, GDM will be diagnosed in accordance with IADPSG criteria: fasting capillary glucose ≥5.1mmol/L and/or 1-h glucose ≥10mmol/L and/or 2-h glucose ≥8.5mmol/L [37]. If a participant develops GDM (by NICE) during the study, she will be asked to continue with the medication and will be referred to the antenatal diabetes team for further advice and treatment. Weight will be measured at this visit.

#### 34–36 weeks’ gestation appointment

Metformin will be dispensed to those allocated the drug. Women will be instructed to stop trial medication on the day of delivery and will be asked to hand in the rest of the trial medication to the research team. Information on any side effects and adverse events will be collected in the metformin arm. Adherence will be assessed by tablet count. Information on pregnancy complications will be collected and weight will be recorded in all arms.

### Process evaluation

A process evaluation will be performed to investigate whether the intervention was implemented as planned and whether participants were satisfied with the intervention. The process evaluation will follow Steckler and Linnan’s framework [38]. This will explore:

1) Context (environmental, socio-economic, political factors)

2) Reach (proportion of target population that participates in the intervention)

3) Dose delivered and dose received (proportion of intervention received)

4) Fidelity (extent to which the intervention was delivered as prescribed by the protocol)

5) Acceptability (if intervention materials and advice were well received by the participants)

Qualitative, semi-structured interviews will be conducted to capture the women’s perceptions and experiences of the study. Women will be recruited (*n*=15, 5 per group) after the intervention has been provided. The interviews will be recorded and transcribed.

### Trial outcomes

#### Primary feasibility outcomes

Trial feasibility will include examination of recruitment, retention, intervention adherence, determination of sample size and acceptability. Recruitment will be determined by calculating the proportion of randomised participants from those screened for eligibility. Retention will be measured by the percentage of participants completing the study through the post-assessment. Intervention attendance will be assessed as the number of diet and physical activity sessions completed, with a range of 0 to 8. Adherence to metformin will be defined as participants taking 80% of the study prescribed medication during the trial as assessed by tablet count. The accelerometer wear time will be reported (hours/day). CGM adherence will be defined by sensor wear ≥ 70%. For future full-scale RCTs using CGM, the sample size will be calculated based on the best estimate of the standard deviation and distribution of mean 24-h glucose as determined in the pilot to detect a 0.5-mmol/L difference between arms. Intervention acceptability will be evaluated by an embedded process evaluation using qualitative research methods. Interview questions will address intervention satisfaction and usefulness in addition to ease of using study devices. Additional questions will address the women’s perceptions of risk and benefits of taking metformin, barriers to adherence and the dose of metformin that is acceptable to women. Feedback on the content of the diet and physical activity components will also be obtained in addition to the delivery format (online delivery and time involved).

#### Secondary patient-centred outcomes

- A reduction in mean glucose/24h of 0.5 mmol/L after 8 weeks of treatment compared to the standard arm as assessed using CGM.
- Other glycaemic parameters after 8 weeks of treatment; mean daytime glucose 0.700–23.00h, mean nocturnal glucose 23.00–0.700h, percentage time in tight glucose control target 3.5–6.7mmol/L, percentage time in recommended glucose control target 3.5–7.8mmol/L, area under the curve (AUC) <6.7mmol/L, AUC <7.8mmol/L, glucose variability measures (standard deviation (SD), coefficient of variation (CV)), high blood glucose index (HBGI) and low blood glucose index (LBGI).
- GDM diagnosis at 24–28 weeks (in accordance with IADPSG and NICE criteria).
- Metabolome; a targeted high-throughput nuclear magnetic resonance (NMR) metabolomic platform (http://computationalmedicine.fi/) will be used to measure metabolites from blood samples at baseline and at the time of the diagnostic OGTT. The platform quantifies lipid measures including lipoprotein particles (VLDL subdivided into 6 subclasses, IDL, LDL subdivided into 3 subclasses and HDL subdivided into 4 subclasses), constituents within each lipoprotein particle type (triglycerides, total cholesterol, free cholesterol and cholesterol ester levels and phospholipid concentrations), fatty acids, amino acids, glycolysis related metabolites, ketone bodies and inflammatory markers.
- Dietary intake and physical activity, including changes in GI, GL, saturated fat, time spent in light and moderate exercise and sedentary behaviour from baseline and after the 8-week intervention.

### Sample size calculation and loss to follow-up

The study is intended as a pilot, one of whose aims is to gather the information needed for planning the definitive trial. This includes estimating the SD of the main outcome (the difference in mean 24-h glucose between arms) and checking the distribution. With twenty participants per arm, the SD can be estimated to within 80% of the true value. A 0.5mmol/L difference in mean 24-h glucose was considered to be of biological relevance based on data from comparable studies of CGM in pregnant women with obesity [39–41]. To account for up to 25% loss to follow-up and achieve a total sample size of 60, a sample of 84 women will be recruited.

### Statistical analysis

An intention-to-treat analysis will be completed for participants in all arms. Demographic characteristics will be compared between groups using Student’s *t* test or Mann–Whitney tests for continuous data and Pearson’s chi-squared test for categorical data as appropriate. For continuous variables, results will be presented as means and standard deviations. The number and percentage will be presented for categorical and binary variables. Following distributional checks, log transformations will be carried out as needed. For the primary and main secondary glycaemic outcomes, an adjustment will be made using multiple linear regression (method analysis of covariance; ANCOVA [42]). For yes/no outcomes, binomial regression will be used with a log link to give risk ratios. For other outcomes, where there is no baseline value, adjustment will be made for the primary outcome only. Analytes measured from a targeted NMR metabolome will be checked for normality; those with non-parametric distribution will be log-transformed. Analyte data at baseline and at the time of the diagnostic OGTT (24–28 weeks) will be compared between women who received the intervention and those who did not. To test for the effect of the intervention on dietary and physical activity outcomes, ANCOVA will be used adjusted for trial entry measurements.

### Data monitoring

A trial steering committee will be established to oversee the conduct and progress of the trial. The terms of reference of the Trial Steering Committee have been developed separately.

All adverse events (AEs) and serious adverse events (SAEs) will be recorded from the time a participant is randomised into the trial. All SAEs, serious adverse reactions (SARs) or unexpected serious adverse reactions (SUSARs) in the lifestyle and metformin arm will be reported immediately by the chief investigator to the King’s Health Partners Clinical Trial Office (KHPs-CTO) in accordance with the current Pharmacovigilance Policy.

### Data management

Types of data collected in this study will include participant information (anonymised) on demography, medical and family history and current pregnancy health which will be collected at study entry. Information on dietary intake, physical activity and glucose homeostasis will be collected pre- and post-intervention. Following delivery, information will be collected from maternal and neonatal medical records regarding health in late pregnancy, labour onset, mode of delivery and inpatient nights. Neonatal outcome data will include admission to special care baby unit. A study-specific Web-based data platform (MedSciNet™) will be used in this trial. All data entries will be logged, providing an effective audit trail including randomisation history. All records will be kept in line with applicable national laws and regulations.

### Confidentiality

All data from maternity records, questionnaires and visit notes will be anonymised; kept confidential; and stored in line with the Medicines for Human Use (Clinical Trials) Amended Regulations 2006 and the General Data Protection Regulation and archived in accordance with the Medicines for Human Use (Clinical Trials) Amended Regulations 2006. The trial database will be locked before the final analysis in line with the local guidelines. All anonymised data will be stored on a password-protected computer with limited access. No personally identifiable information will be included in any publications or presentations relating to this study.

## Discussion

Several RCTs including UPBEAT have attempted to improve pregnancy outcomes using lifestyle and/or pharmacological intervention in pregnant women with obesity. The lack of success of these RCTs in unselected women has shifted the focus to targeted interventions for those individuals identified at greatest risk of adverse pregnancy outcomes. Early identification and treatment of high-risk women may improve the health of the mother and her infant.

This study is designed to provide evidence that early risk stratification of pregnant women with obesity for later development of GDM and early targeted intervention using lifestyle advice with or without metformin is feasible and improves glucose tolerance compared to standard antenatal care. If the study proves effective, the next stage will be the development of a protocol for an RCT adequately powered for examining a reduction in the prevalence of GDM. The aim will be to determine, whether in a high-risk subgroup of pregnant women with obesity, targeted intervention with lifestyle advice +/− metformin is an effective strategy for the prevention of GDM.

## Data Availability

Not applicable.

## Abbreviations

AEs: Adverse events
AUC: Area under the curve
BMI: Body mass index
CGM: Continuous glucose monitoring
CV: Coefficient of variation
FFQ: Food frequency questionnaire
GDM: Gestational diabetes mellitus
GI: Glycaemic index
GL: Glycaemic load
HBGI: High blood glucose index
IADPSG: The International Association of Diabetes and Pregnancy Study Groups
KHPs-CTO: King’s Health Partners Clinical Trial Office
LBGI: Low blood glucose index
LGA: Large-for-gestational age
NHS: The National Health Service
NICE: The National Institute of Clinical Excellence
NMR: Nuclear magnetic resonance
OGTT: Oral glucose tolerance test
RCT: Randomised controlled trial
SAEs: Serious adverse events
SARs: Serious adverse reactions
SD: Standard deviation
SUSARs: Unexpected serious adverse reactions
